# Cellular Immune Response to BNT162b2 mRNA COVID-19 Vaccine in a Large Cohort of Healthcare Workers in a Tertiary Care University Hospital

**DOI:** 10.3390/vaccines10071031

**Published:** 2022-06-27

**Authors:** Cristina Costa, Gitana Scozzari, Enrica Migliore, Claudia Galassi, Giovannino Ciccone, Guido Ricciardelli, Antonio Scarmozzino, Lorenzo Angelone, Paola Cassoni, Rossana Cavallo, on behalf of the Collaborative Group

**Affiliations:** 1 Microbiology and Virology Unit, University Hospital Città Della Salute e Della Scienza di Torino, 10126 Turin, Italy; guido.ricciardelli@unito.it (G.R.); rossana.cavallo@unito.it (R.C.); 2 Department of Public Health and Pediatric Sciences, University of Turin, 10126 Turin, Italy; 3 Molinette Hospital Medical Direction, University Hospital Città Della Salute e Della Scienza di Torino, 10126 Turin, Italy; gscozzari@cittadellasalute.to.it (G.S.); ascarmozzino@cittadellasalute.to.it (A.S.); langelone@cittadellasalute.to.it (L.A.); 4 Clinical Epidemiology Unit, University Hospital Città Della Salute e Della Scienza di Torino and CPO Piemonte, 10126 Turin, Italy; emigliore@cittadellasalute.to.it (E.M.); cgalassi@cittadellasalute.to.it (C.G.); gciccone@cittadellasalute.to.it (G.C.); 5 Pathology Unit, Department of Medical Sciences, University of Turin, 10126 Turin, Italy; paola.cassoni@unito.it

**Keywords:** SARS-CoV-2, immunity, cellular, mRNA vaccination, health care workers

## Abstract

We describe the results of a T-cell immunity evaluation performed after a median elapsed time of 7 months from second-dose BNT162b2 vaccine administration, in a representative sample of 419 subjects from a large cohort of hospital workers. Overall, the Quantiferon SARS-CoV-2 assay detected a responsive pattern in 49.9%, 59.2% and 68.3% of subjects to three different antigenic stimuli from SARS-CoV-2, respectively, with 72.3% of positivity to at least one antigenic stimulus. Potential predictors of cellular response were explored by multivariable analyses; factors associated with positivity to cellular response (to Ag1 antigenic stimulus) were a previous SARS-CoV-2 infection (OR = 4.24, 95% CI 2.34–7.67, *p* < 0.001), increasing age (per year: OR = 1.03 95% CI 1.01–1.06, *p* = 0.019 and currently smoking (compared to never smoking) (OR = 1.93, 95% CI 1.11–3.36, *p* = 0.010). Increasing time interval between vaccine administration and T-cell test was associated with decreasing cellular response (per week of time: OR = 0.94, 95% CI 0.91–0.98, *p* = 0.003). A blood group A/AB/B (compared to group O) was associated with higher levels of cellular immunity, especially when measured as Ag2 antigenic stimulus. Levels of cellular immunity tended to be lower among subjects that self-reported an autoimmune disorder or an immunodeficiency and among males. Further studies to assess the protective significance of different serological and cellular responses to the vaccine toward the risk of reinfection and the severity of COVID-19 are needed to better understand these findings.

## 1. Introduction

The BNT162b2 vaccine (Comirnaty^®^, Pfizer/BioNTech, Mainz, Germany) is a lipid nanoparticle–formulated, nucleoside-modified RNA (modRNA) encoding the SARS-CoV-2 full-length spike glycoprotein, with an efficacy in preventing COVID-19 approaching 95% [1]. Studies in experimental models and humans evidenced that BNT162b2 vaccine elicits both high levels of neutralizing antibodies, targeting the receptor-binding domain (RBD) and strong Th1-skewed functional CD4+ and CD8+ T-lymphocyte responses [2,3]. Similarly, several studies on COVID-19 patients demonstrated the SARS-CoV-2 induction of specific antibodies and T cell responses able to control viral replication and to reduce disease severity [4]. Cytotoxic T cells and Th1 cells play a crucial role after immunization with mRNA vaccines: Th1 lymphocytes are primed following the presentation of the spike protein by dendritic cells, whereas cytotoxic T cells are activated upon the recognition of the viral protein as an endogenous antigen bound to the HLA-I molecule and presented on the cell surface. Among the different methods to evaluate T-cell responses to SARS-CoV-2 vaccination there are interferon-gamma releasing assays (IGRA), that provide a quantitative measure of cellular immune response as levels of specifically secreted IFN-γ and are used in other clinical contexts, such as transplantation [5]. Currently, the literature on the dynamics and magnitude of cellular response in vaccinated individuals is still limited [6,7,8], particularly regarding the duration of T-cell responses after vaccination [9,10,11,12,13] and the factors that could explain the interindividual heterogeneity in immunological response [14].

We previously reported, in a large population of health care workers (HCW), the serological response at 4–5 months post-BNT162b2 administration, which was present in up to 99% of subjects [15]. Herein, we report the SARS-CoV-2 specific T-cell responses in a random sample of these subjects at a median of 7.2 months following vaccination, using a commercially available test, and explorative analyses on factors potentially influencing the magnitude and duration of the cellular immune response (including prior SARS-CoV-2 exposure).

## 2. Materials and Methods

### 2.1. Study Design and Population

The study was conducted at the University Hospital Città della Salute e della Scienza di Torino (CSS), the largest tertiary care Public Utility in northwestern Italy. The study design was extensively described previously [15,16]. Briefly, this cohort study firstly evaluated the SARS-CoV-2 IgG seropositivity among 8769 CSS workers after the first pandemic wave in northern Italy [16]. Thereafter, the protocol was expanded in order to evaluate the immune response (both humoral and cellular mediated) after COVID-19 vaccination. Since 27 December 2020, COVID-19 vaccines (the large majority being the BNT162b2 mRNA vaccine) were offered to all CSS workers and vaccinations took place mostly in January–February 2021. During the month of May 2021, all CSS workers were invited to attend the immune response post-vaccination survey; 6687 vaccinated subjects adhered by filling in the anamnestic questionnaire and supplying the blood sample for the IgG assay [15]. 

Subsequently, in a random subset of workers among those attending the serological survey, we evaluated the cellular immune response. To this purpose, we randomly extracted a sample of 550 vaccinated subjects, stratified by levels (quintiles) of serological IgG values (1° quintile: ≤475 BAU/mL; 2°quintile: 476–789 BAU/mL; 3° quintile: 790–1250 BAU/mL; 4° quintile: 1251–2079 BAU/mL and 5° quintile: ≥2080 BAU/mL) [15]. In the lowest quintile we also included nine subjects with a previous negative post vaccination serological test who agreed to attend the study. Subjects who accepted participation were asked to give a blood sample for the cellular test between July and October 2021. No subject received a third dose of vaccine before participating in any of the tests we report here.

All the subjects signed an informed consent. The study protocol was approved by CSS Institutional Review Board (protocol n. 0046457, 27 April 2021).

### 2.2. Evaluation of SARS-CoV-2 Specific Immune Responses

Serological data on serum specimens were studied by the LIAISON^®^ SARS-CoV-2 Trimeric IgG assay chemiluminescent immunoassay (CLIA) (Diasorin, Saluggia, Italy), following the manufacturer’s instructions and using the LIAISON^®^ XL Analyzer [17], as previously described [15]. Antibody concentrations were expressed as binding antibody units (BAU/mL) and calibrated with the First WHO International Standard for anti-SARS-CoV-2 immunoglobulin NIBSC code 20/136 [18], allowing for a qualitative grading of the results: <33.8 BAU/mL considered as negative; ≥33.8 BAU/mL as positive. The upper limit for the quantification of antibody levels, with no further sample dilution, was 2080 BAU/mL, while the lower detection limit was 4.81 BAU/mL.

The T-cell response was evaluated using the Quantiferon SARS-CoV-2 test (Qiagen, Milan. Italy), an interferon-γ releasing assay (IGRA) that consists of three antigen tubes, SARS-CoV-2 Ag1, Ag2 and Ag3, which use a combination of proprietary specific antigen peptides to stimulate lymphocytes involved in cell-mediated immunity in heparinized whole blood. The Quantiferon SARS CoV-2 Ag1 tube contains CD4+ epitopes derived from the S1 subunit (Receptor Binging Domain) of the Spike protein, the Ag2 tube contains CD4+ and CD8+ epitopes from the S1 and S2 subunits of the Spike protein and the Ag3 tube consists of CD4+ and CD8+ epitopes from S1 and S2, plus immunodominant CD8+ epitopes derived from whole genome [9]. Specimens were processed following the manufacturer’s instruction. Briefly, venous blood specimens were collected directly into the Quatiferon tubes containing the above-mentioned specific peptides as well as negative (nil tube) and positive (mitogen tube) controls. Whole blood was incubated at 37 °C for 16–24 h and centrifuged to separate plasma. IFN-γ was measured in plasma using ELISA test and expressed as IU/mL. For quantification, the nil tube value was substracted to mitigate against the background; a reactive response was considered for levels ≥ 0.15 IU/mL, according to the manufacturer’s instructions (the assay is CE-IVD intended for Ag1 and Ag2, whereas this research used only Ag3).

### 2.3. Statistical Analysis

The baseline characteristics of participants and the results of immunological evaluations (Ag positivity) are summarized as absolute and relative (percentage) frequencies. Quantitative variables with non-normal distribution (including Ag levels) are reported as medians and interquartile ranges (IQR). Correlations between Ag were performed using log-transformed Ag values. To explore the potential predictors of cellular immune response after vaccination, we performed different multivariable analyses for each Ag, applying three methods to define the outcome: (a) ordinal logistic regression models using as outcome the quintiles of each Ag distribution (Table S1), considering the lowest category as reference; the estimated Odds ratios (OR), with 95% confidence intervals (95% CI), expressing the risk of belonging to a higher quintile of the Ag distribution, after accounting for all other variables; (b) regression models using the log transformed Ag values as outcomes; (c) logistic regression models using the percentage of Ag to positivity apply the cut-off ≥ 0.15 IU/mL.

Information on demographic and clinical characteristics were obtained from questionnaires filled in by subjects. Time elapsed between vaccination and T-cell response test was calculated as the number of weeks (or months) between the date of the second vaccine dose and the date of the T cell test. Previous exposure to SARS-CoV-2 was defined as previously described [15].

Statistical analyses were performed by Stata 15.1 software (StataCorp LP, College Station, TX, USA).

## 3. Results

**Figure 1:**
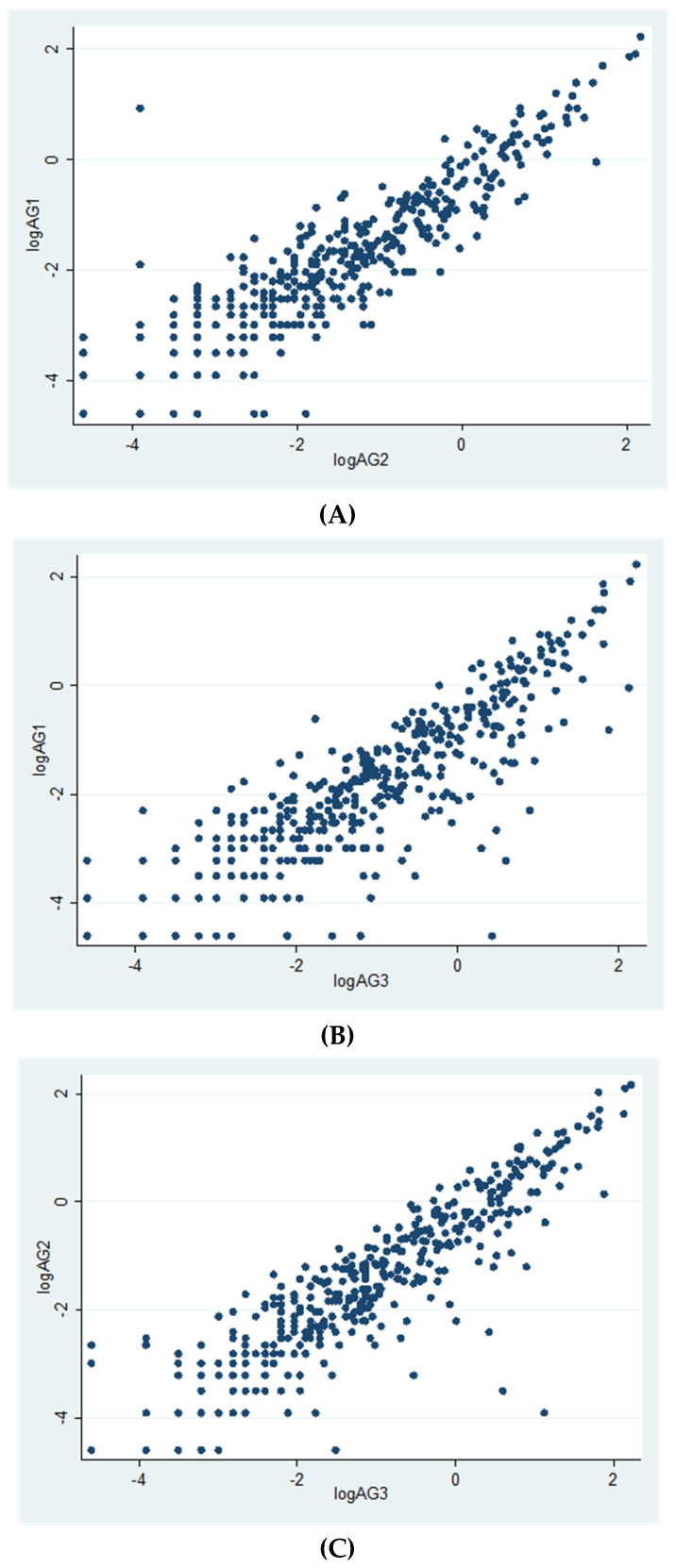
Correlations between Ag values (log-transformed). (**A**): logAg1 vs. logAg2 (r = 0.90, *p* = 0.0000); (**B**): logAg1 vs. logAg3 (r = 0.85, *p* = 0.0000); (**C**): logAg2 vs. logAg3 (r = 0.89, *p* = 0.0000).

Overall, 419 subjects (representing 76.2% of the random sample) attended the study (males/females 86/333; median age 51.0 years, IQR 45.1–56.9). All the subjects received both doses of the vaccine (BNT162b2 mRNA vaccine), mostly in January–February 2021, and attended the post-vaccination serological survey performed in May 2021. Cellular immunity was evaluated at a median time elapsed of 31.3 weeks (IQR 25–34.1 weeks) from vaccination; 64% of subjects were evaluated at 6 months or more after second dose vaccination (Table 1; range 2.4–9.2 months). Overall, prevalence of positivity was 49.9% for Ag1, 59.2% for Ag2 and 68.3% for Ag3 (Table 1), and 72.3% of individuals responded to at least one Ag; median values were 0.14, 0.20 and 0.32 IU/mL for Ag1, Ag2 and Ag3, respectively (Table 2). Correlations among the three Ag were very high (Figure 1).

Table 1 reports the prevalence of cellular immune reactivity to the three different antigenic stimuli in relation to main demographic, occupational and clinical features, while the median Ag values for the same features are reported in Table 2; a large variability in median Ag values was observed for each of the subjects’ characteristics considered (Table 2).

To explore the role of single predictive factors on the cellular response we performed multivariable analyses; we used different methods to evaluate the intensity of cellular response: (a) risk of belonging to a higher quintile (Table S1) of the Ag distributions; (b) risk of having higher log-transformed Ag values and c) risk of having a positive Ag value (>= 0.15 IU/mL). The complete results are reported in Table 3, Table 4 and Table 5, while Figures S1–S3 reported in the Supplementary Materials summarize the results of the associations for the quintiles of each Ag. Overall, for most of the investigated factors the directions of the associations were consistent both among the different statistical methods used to define the outcome for each Ag and also among the three different antigenic stimuli (Table 3, Table 4 and Table 5).

Increasing age was consistently associated with a higher Ag response, for all Ag, significant for quintiles of Ag3 (OR 1.02, 95% IC 1.00–1.04, *p* = 0.027 for each year of age) as well as for the positivity of all the Ag (Table 3, Table 4 and Table 5). Reactivity tended to be lower in males compared to females, with clearer results for Ag1 and Ag3 positivity (Table 3, Table 4 and Table 5). Currently smoking (compared to never smoking) was consistently associated with higher reactivity for all Ag, especially for Ag1 and Ag2 (Table 3, Table 4 and Table 5).

We observed a higher Ag3 cellular response among subjects with overweight/obesity, particularly when measured as Ag3 positivity (Table 5), while results are much less consistent for Ag2 and Ag1 (Table 3 and Table 4). Blood group A/B/AB (compared to blood group O) was strongly associated with a higher Ag response, for each of the three different antigenic stimuli and particularly for Ag2. Unexpectedly, among subjects who did not report the blood group we observed an inverse association. A previous SARS-CoV-2 infection (at any time between March 2020 and May 2021) was strongly and consistently associated with higher reactivity for all Ag, with an OR of about 3 (Table 3, Table 4 and Table 5, Figures S1–S3). The self-reporting of an auto-immune disease was consistently associated with a lower T-cell response for all the Ag, with significant results for Ag1 and Ag3 positivity (Table 3, Table 4 and Table 5). A self-reporting of an immunodeficiency was also related to a lower Ag response, for all the Ag, although with very large confidence intervals and with some inconsistencies between the outcome measures, possibly due to the small number of subjects (n.15) reporting this condition. A higher T-cell response seems related to the self-reporting of diabetes, but the uncertainty of the estimates is very large (only 10 subjects reported the disease) and preclude firm conclusion. Some differences of AG responses were also observed among different job profiles, without a clear pattern. Finally, we observed a consistent, significant inverse association between all Ag response and time elapsed between vaccination and T-cell test (f.i., for Ag1 quintiles, OR = 0.96, 95%IC 0.93–0.99 for each week of increasing distance).

Table S2 graphically summarizes the directions of the associations between some individual characteristics and post-vaccination T-cell response observed in the present study, in comparison with those previously observed between the same factors and post-vaccination serological values [15].

## 4. Discussion

In this study, we describe the results of T-cell immunity evaluation mostly performed over a period between 5 and 9 months post-vaccination in a random sample of 419 hospital workers of our large cohort, in order to evaluate cellular immune response to BNT162b2 vaccine. Using the Quantiferon SARS-CoV-2 assay, at a median elapsed time of more than 7 months from second-dose vaccine administration, we found a responsive pattern in 49.9%, 59.2% and 68.3% of subjects to Ag1, Ag2 and Ag3, respectively, and 72.3% of individuals responded at least to one Ag. At our knowledge, this is the first study that evaluated T-cell immunity response and explored its potential predictors in a large, representative sample of real-world hospital workers, at a median time elapsed greater than 7 months after second-dose vaccine administration. Few studies have evaluated T-cell responses after two doses of the BNT162b2 vaccine and mainly focused on recently vaccinated individuals (i.e., first two months post-vaccination) or on smaller series [19]; higher rates of T-cell responsiveness compared to those observed in our study were often observed [20,21,22], similar to the rates reported for humoral responses [9], a finding consistent with a decline in T-cell mediated responses over time observed by others [10,12,23] as well as in the present study. However, it is noteworthy that in our study a cellular response ≥ 0.15 IU/mL persists up to 7 months post-vaccination in more than 60–70% of subjects (depending on the Ag), also among subjects without previous SARS-CoV-2 infection (Table S3). Data on immune memory to SARS-CoV-2 are currently limited: in a study on cellular immunity >6 months post-infection, memory CD4+ and CD8+ cells were positive in approximately 90% and 70% of individuals, respectively [24]. Long-lasting cellular immunity has been described in convalescent plasma from individuals up to 11 months post-infection, as well as in previously infected subjects after one dose of vaccine [25].

We observed higher responsiveness in Ag3 tubes than in Ag2 and in Ag2 than in Ag1, considering both the rate and amount of response (median and IQR 0.14 IU/mL [0.05–0.4], 0.2 [0.07–0.61], 0.32 [0.11–0.9], respectively). Accordingly to overlapping findings from others [9,26], the higher response in Ag2 compared to Ag1 tubes underlines that both CD4+ and CD8+ T cells contribute to the T-cell responses and that robust CD4+ and CD8+ responses are elicited by the BNT162b2 vaccine [2].The Ag3 tube (consisting of a selection of immunodominant peptides) evidenced the highest response in terms of responsiveness and of IFN-γ levels in most individuals, including subjects without previous SARS-CoV-2 infection, differently from what was expected. Other studies reported this finding in some individuals only, hypothesizing that this could be due to the selection of peptides included in the Ag3 tube from the whole SARS-CoV-2 genome, as all subjects received spike-based vaccines [9]. Thus, a potential effect from pre-existing cross-reactive immune responses against endemic human coronaviruses cannot be excluded. SARS-CoV-2 is relatively distant related to four endemic human coronaviruses (HCoV-OC42, HCoV-HKU1, HCoV-229E and HCoV-NL63 beta- and alphacoronaviruses), which cause the common cold, with a <10% aminoacidic identity in the spike receptor-binding domain. Therefore, a cross-reactive humoral response anti-spike appears to be rare [6,27,28,29,30]. In contrast, a cross-reactive T cell memory appears to be relevant, seen in up to 28–50% of people [6,31,32,33,34], the majority of which being CD4+ T cells, although CD8+ T cells have also been observed [35,36]. The occurrence and role of cross-reactive memory T cells is quite intriguing as it relates to COVID-19 vaccines, as people with some degree of pre-existing immunity may respond differently to vaccines than people without such T cell memory. One limitation is that the Quantiferon SARS-CoV-2 assay for Ag 1, Ag, 2 and Ag3 does not allow the distinction between vaccine-elicited and infection-elicited cellular immune responses. It could be hypothesized that a test-evaluating response to selected immunodominant epitopes from the whole SARS-CoV-2 genome, except for the spike protein, would be useful in this context.

For each Ag, we observed a wide spectrum of IFN-γ values for each of the subjects’ characteristics considered, including previous serological values. A large variability in the magnitude of T cell responses after vaccination has been reported [7,9,26], with weak [37,38,39] or absent [26] correlations between antibody values and IFN-γ levels; in the study by Angyal, a weak association was found among SARS-CoV-2-naïve HCW, but not among SARS-CoV-2-experienced HCW [25]. It is important to note that in our study serology was performed at a median of 4 months post-vaccination, whereas cellular immunity was evaluated mostly between 5 and 9 months post-vaccination, due to the organization of the specimen collection, making it unrealistic to compare serology and cellular immunity.

We explored the possible predictors of cellular response intensity by means of multivariable models, focusing on demographic, individual and clinical factors already investigated as the possible predictors of humoral response in our large cohort of vaccinated HCW [15].

As expected, and in analogy to serological responses, previous SARS-CoV-2 infection was the main predictor of responsiveness and levels of IFN-γ after vaccination, with an increased risk of about three times. In a large study on T-cell and antibody responses to the first BNT162b2 vaccine dose in UK health care workers, spike-specific T-cell responses one month following first vaccine dose were 5.2 times higher in previously infected subjects than in SARS-CoV-2-naïve individuals [25]. Similar findings have been observed in several other studies [40,41,42,43]. These observations suggested single vaccination regimens in COVID-19-recovered individuals are beneficial, particularly in periods or countries with scarce vaccine supplies [44]. In the SWITCH trial on a population of 434 health care workers receiving a priming dose of the Ad26.COV2.S (Janssen) vaccine, both antibody and IFN-γ levels significantly increased 28 days after a homologous or heterologous booster vaccination, in comparison to no booster [45]. In our study, all subjects received two doses; therefore, the difference between subjects with and without previous infection underlines the potential increment in T-cell immunity was determined by the combination of complete vaccination and infection.

Regarding demographic features, we found a trend to a low level of responsiveness in the male gender, also when adjusted for all other variables, as for serology. Other authors did not observe a gender effect [12,23,38,39]; there is scarceness of literature data on this issue, and this finding should be re-evaluated with more relevant evidence from other studies. For SARS-CoV-2 infection and severity, a role has been proposed for encoding on the X-chromosome of the angiotensin-converting enzyme 2 and the largest immune-related genes leading females to develop more robust immune responses, as well as a role for sex hormones [46].

We previously documented a clear inverse association between age and humoral response in our cohort of vaccinated HCW, consistent with literature data [15]; on the other hand, in this study, cellular immunity in terms of responsiveness and level of response was directly correlated with increasing age; other studies performed on HCW found non-significant reduction in T-cell response with increasing age [12,25,38] or even a vaccine-induced CD8+ T cell response enhanced in older males [23]. Although our observation deserves further evaluation, it is likely that pre-existing cross-reactive immune responses could play a role, as previously discussed.

We found that smoking habits were associated with higher T-cell response in vaccinated HCW, even after adjustment for all other factors. We do not have explanations for this finding, which is apparently in contrast with previous observations of a reduced humoral response both after SARS-CoV-2 infection [16] and after vaccination [15] among smokers in our cohort of HCW. To the best of our knowledge, the literature on this topic is at present lacking, and this finding is without a clear interpretation.

Another finding is the association between blood groups other than O and a higher level of responsiveness, in contrast to serology. Some studies suggested a relation between blood groups and COVID-19 infection and severity [47]. The Severe COVID-19 GWAS Group identified a gene cluster in patients with COVID-19 and confirmed a potential involvement of the ABO blood type, with a higher risk in blood type A and a protective role in type O [48]. The impact of ABO blood type on vaccine immunogenicity in terms of humoral and cellular response is unclear and further studies are certainly needed.

Considering clinical features, the self-reported occurrence of immunodeficiency tended to be associated, although not significantly, with low responsiveness and level of IFN-γ production, as already seen for serology. Similarly, the self-reported occurrence of autoimmune diseases also tended to be associated to lower levels of cellular immunity, whereas no significant association was found for serology in our previous study [15]. Although we have to consider the limit of self-reporting, it is likely that impairment in immune responses in immune-mediated diseases, as well as specific therapies, may play a relevant role in vaccine immunogenicity, as already described for example in multiple sclerosis and immune-mediated inflammatory rheumatic diseases [49,50,51,52,53] or immunodeficiency [54,55].

Methods used to study cellular immunity deserve some comments. There are different methods to evaluate T-cell responses to SARS-CoV-2, such as flow cytometry and IGRA assays, including Quantiferon and ELISPOT-assay. Some of these methods have limitations in the clinical use compared to whole blood IGRA assays, whereas overlapping data on sensibility and specificity have been found for methods by SARS-CoV-2 IGRA-type chemiluminescence and ELISA [38]. In this study, we employed an IGRA to evaluate cellular immune response. This type of method proved to be a valid tool to define vaccine responsiveness [56], due to the fundamental T cell engagement following vaccination as well as COVID-19 cases, irrespective of the severity of the disease [57].

Overall, studies on cellular immunity for routine use in a clinical laboratory underline the usefulness of a complementary correlate of protection in addition to serology, in particular in some groups, such as health care workers, and other individuals with risk factors regarding exposure, e.g., immunosuppressed patients, such as transplant recipients who may fail to mount a measurable antibody response.

## 5. Conclusions

In this study, we evaluated cellular immune response and explored its potential predictors in a large and representative sample of our cohort of HCW after roughly 7 months from second-dose BNT162b2 vaccine administration. We observed that some factors influenced cellular response in the same way as they did humoral response (i.e., a previous SARS-CoV-2 infection and time elapsed between vaccination and immunity test); conversely, we observed the opposite pattern for other factors, such as age (with increasing cellular response with increasing age) or active smoking (associated with increasing cellular response). Further studies to assess the protective significance of different serological and cellular responses to the vaccine regarding both the risk of reinfection and the clinical manifestations of COVID-19 are needed to better understand these findings.

## Figures and Tables

**Table 1:** Subjects included in the study (second column from the left) and prevalence of positivity for each antigenic stimuli (Ag) (three rightmost columns) among 419 vaccinated HCW of CSS, by subjects’ characteristics.

	Subjects	Ag1 Positivity	Ag2 Positivity	Ag3 Positivity
	N (Column %)	N (Row %)	N (Row %)	N (Row %)
**OVERALL**	419	209 (49.9%)	248 (59.2%)	286 (68.3%)
**Age**				
Median (Q1, Q3)	51.0 (45.1, 56.9)	51.5 (46.9, 57.3)	51.3 (46.2, 57.1)	51.6 (46.2, 57.3)
**Age groups**				
≤29	23 (5.5%)	5 (21.7%)	8 (34.8%)	9 (39.1%)
30–39	47 (11.2%)	18 (38.3%)	23 (48.9%)	29 (61.7%)
40–49	120 (28.6%)	66 (55.0%)	78 (65.0%)	86 (71.7%)
50–59	180 (43.0%)	92 (51.1%)	108 (60.0%)	124 (68.9%)
≥60	49 (11.7%)	28 (57.1%)	31 (63.3%)	38 (77.6%)
**Gender**				
Female	333 (79.5%)	172 (51.7%)	197 (59.2%)	233 (70.0%)
Male	86 (20.5%)	37 (43.0%)	51 (59.3%)	53 (61.6%)
**Job profile**				
Nurse	163 (38.9%)	72 (44.2%)	90 (55.2%)	102 (62.6%)
Administrative staff	46 (11.0%)	24 (52.2%)	31 (67.4%)	35 (76.1%)
IT/maintenance staff	23 (5.5%)	14 (60.9%)	14 (60.9%)	17 (73.9%)
Physician	76 (18.1%)	41 (53.9%)	47 (61.8%)	54 (71.1%)
Health care assistant (HCA)	33 (7.9%)	20 (60.6%)	19 (57.6%)	24 (72.7%)
Clinical staff (other than physician/nurse/HCA)	78 (18.6%)	38 (48.7%)	47 (60.3%)	54 (69.2%)
**Smoking habits**				
Former smokers	75 (17.9%)	40 (53.3%)	46 (61.3%)	49 (65.3%)
Never smokers	258 (61.6%)	121 (46.9%)	145 (56.2%)	178 (69.0%)
Current smokers	86 (20.5%)	48 (55.8%)	57 (66.3%)	59 (68.6%)
**BMI**				
Underweight (BMI < 18.5)	14 (3.3%)	7 (50.0%)	8 (57.1%)	8 (57.1%)
Normal weight (BMI18.5–25)	255 (60.9%)	122 (47.8%)	144 (56.5%)	165 (64.7%)
Overweight (BMI 25–30)	101 (24.1%)	53 (52.5%)	62 (61.4%)	74 (73.3%)
Obesity (BMI > 30)	49 (11.7%)	27 (55.1%)	34 (69.4%)	39 (79.6%)
**Blood group**				
O	158 (37.7%)	75 (47.5%)	83 (52.5%)	106 (67.1%)
A/B/AB	195 (46.5%)	107 (54.9%)	135 (69.2%)	141 (72.3%)
Unknown	66 (15.8%)	27 (40.9%)	30 (45.5%)	39 (59.1%)
**Previous SARS-CoV-2 infection**				
No	337 (80.4%)	148 (43.9%)	186 (55.2%)	217 (64.4%)
Yes	82 (19.6%)	61 (74.4%)	62 (75.6%)	69 (84.1%)
**Time elapsed (months) between vaccination and T-cell test**				
<5	26 (6.2%)	15 (57.7%)	17 (65.4%)	18 (69.2%)
≥5–<6	126 (30.1%)	73 (57.9%)	79 (62.7%)	95 (75.4%)
≥6–<7	42 (10.0%)	27 (64.3%)	30 (71.4%)	31 (73.8%)
≥7–<8	136 (32.5%)	65 (47.8%)	77 (56.6%)	90 (66.2%)
≥8	89 (21.2%)	29 (32.6%)	45 (50.6%)	52 (58.4%)
**Quintiles of serological value**				
1st (lowest)	87 (20.8%)	34 (39.1%)	45 (51.7%)	50 (57.5%)
2nd	81 (19.3%)	32 (39.5%)	39 (48.1%)	51 (63.0%)
3rd	75 (17.9%)	36 (48.0%)	48 (64.0%)	48 (64.0%)
4th	92 (22.0%)	53 (57.6%)	57 (62.0%)	69 (75.0%)
5th	84 (20.0%)	54 (64.3%)	59 (70.2%)	68 (81.0%)
**Autoimmune diseases**				
No	359 (85.7%)	184 (51.3%)	218 (60.7%)	254 (70.8%)
Yes	60 (14.3%)	25 (41.7%)	30 (50.0%)	32 (53.3%)
**Immuno-deficiency**				
No	404 (96.4%)	203 (50.2%)	241 (59.7%)	278 (68.8%)
Yes	15 (3.6%)	6 (40.0%)	7 (46.7%)	8 (53.3%)
**Hypertension**				
No	346 (82.6%)	172 (49.7%)	205 (59.2%)	237 (68.5%)
Yes	73 (17.4%)	37 (50.7%)	43 (58.9%)	49 (67.1%)
**Diabetes**				
No	409 (97.6%)	204 (49.9%)	242 (59.2%)	278 (68.0%)
Yes	10 (2.4%)	5 (50.0%)	6 (60.0%)	8 (80.0%)
**Allergic rhinitis**				
No	345 (82.3%)	173 (50.1%)	203 (58.8%)	236 (68.4%)
Yes	74 (17.7%)	36 (48.6%)	45 (60.8%)	50 (67.6%)
**Respiratory diseases**				
No	403 (96.2%)	199 (49.4%)	238 (59.1%)	275 (68.2%)
Yes	16 (3.8%)	10 (62.5%)	10 (62.5%)	11 (68.8%)
**Neoplasms**				
No	403 (96.2%)	200 (49.6%)	240 (59.6%)	277 (68.7%)
Yes	16 (3.8%)	9 (56.3%)	8 (50.0%)	9 (56.3%)
**Other chronic diseases**				
No	409 (97.6%)	205 (50.1%)	241 (58.9%)	278 (68.0%)
Yes	10 (2.4%)	4 (40.0%)	7 (70.0%)	8 (80.0%)

**Table 2:** Levels of each antigenic stimuli (Ag, median and interquartile range—IQR), by subjects’ characteristics.

	Ag1 IU/mL	Ag2 IU/mL	Ag3 IU/mL
	Median (IQR)	Median (IQR)	Median (IQR)
**OVERALL**	**0.14 (0.05–0.4)**	**0.20 (0.07–0.61)**	**0.32 (0.11–0.92)**
**Age groups**			
≤29	0.06 (0.03–0.13)	0.11 (0.05–0.20)	0.13 (0.06–0.31)
30–39	0.11 (0.06–0.33)	0.14 (0.08–0.50)	0.23 (0.11–0.64)
40–49	0.17 (0.08–0.42)	0.24 (0.08–0.78)	0.35 (0.13–1.14)
50–59	0.15 (0.05–0.46)	0.23 (0.06–0.66)	0.34 (0.11–1.10)
≥60	0.18 (0.08–0.37)	0.23 (0.12–0.61)	0.32 (0.16–0.87)
**Gender**			
Female	0.15 (0.06–0.40)	0.20 (0.07–0.59)	0.32 (0.12–0.92)
Male	0.13 (0.04–0.40)	0.21 (0.05–0.67)	0.32 (0.07–0.84)
**Job profile**			
Nurse	0.11 (0.05–0.38)	0.17 (0.06–0.61)	0.28 (0.09–1.00)
Administrative staff	0.15 (0.06–0.49)	0.25 (0.06–0.80)	0.49 (0.15–1.16)
IT/maintenance staff	0.16 (0.05–0.25)	0.26 (0.08–0.66)	0.41 (0.10–0.84)
Physician	0.16 (0.06–0.31)	0.21 (0.11–0.42)	0.32 (0.13–0.70)
Health care assistant (HCA)	0.20 (0.06–0.51)	0.23 (0.05–0.58)	0.28 (0.12–0.64)
Clinical staff (other than physician/nurse/HCA)	0.14 (0.07–0.44)	0.18 (0.09–0.62)	0.3 (0.11–1.12)
**Smoking habits**			
Former smokers	0.15 (0.06–0.42)	0.26 (0.06–0.97)	0.41 (0.09–1.35)
Never smokers	0.13 (0.05–0.34)	0.17 (0.07–0.51)	0.30 (0.11–0.88)
Current smokers	0.19 (0.06–0.42)	0.24 (0.08–0.70)	0.34 (0.12–0.87)
**BMI**			
Underweight (BMI < 18.5)	0.15 (0.05–0.27)	0.17 (0.09–0.48)	0.23 (0.11–0.93)
Normal weight (BMI18.5–25)	0.13 (0.05–0.40)	0.17 (0.07–0.61)	0.28 (0.10–0.85)
Overweight (BMI 25–30)	0.17 (0.05–0.41)	0.21 (0.07–0.55)	0.36 (0.12–1.00)
Obesity (BMI > 30)	0.16 (0.08–0.47)	0.29 (0.10–0.73)	0.38 (0.20–0.94)
**Blood group**			
O	0.13 (0.06–0.31)	0.16 (0.07–0.52)	0.29 (0.11–0.80)
A/B/AB	0.18 (0.07–0.46)	0.29 (0.01–0.72)	0.41 (0.12–1.32)
Unknown	0.08 (0.04–0.25)	0.13 (0.04–0.42)	0.17 (0.06–0.72)
**Previous SARS-Cov-2 infection**			
No	0.11 (0.05–0.30)	0.17 (0.06–0.48)	0.27 (0.09–0.73)
Yes	0.28 (0.13–1.04)	0.43 (0.16–1.41)	0.83 (0.28–2.03)
**Time elapsed (months) between vaccination and IC QTF test**			
<5	0.16 (0.08–0.40)	0.24 (0.06–1.17)	0.37 (0.11–1.85)
≥5–<6	0.20 (0.07–0.47)	0.30 (0.10–0.70)	0.39 (0.15–1.32)
≥6–<7	0.31 (0.08–0.68)	0.42 (0.11–1.21)	0.50 (0.13–1.37)
≥7–<8	0.13 (0.05–0.32)	0.17 (0.07–0.52)	0.28 (0.10–0.83)
≥8	0.09 (0.04–0.20)	0.15 (0.05–0.31)	0.21 (0.07–0.54)
**Quintiles of serological value**			
1st (lowest)	0.07 (0.03–0.21)	0.15 (0.02–0.40)	0.20 (0.07–0.59)
2nd	0.11 (0.05–0.26)	0.14 (0.07–0.33)	0.21 (0.10–0.67)
3rd	0.13 (0.05–0.34)	0.22 (0.06–0.66)	0.31 (0.09–0.77)
4th	0.18 (0.07–0.61)	0.24 (0.08–0.94)	0.44 (0.15–1.35)
5th	0.25 (0.10–0.73)	0.35 (0.13–1.04)	0.59 (0.27–1.75)
**Autoimmune diseases**			
No	0.15 (0.06–0.40)	0.21 (0.08–0.61)	0.32 (0.11–0.90)
Yes	0.08 (0.03–0.40)	0.15 (0.04–0.66)	0.18 (0.06–1.28)
**Immuno-deficiency**			
No	0.15 (0.06–0.41)	0.21 (0.07–0.62)	0.32 (0.11–0.93)
Yes	0.07 (0.01–0.22)	0.09 (0.02–0.31)	0.15 (0.04–0.67)
**Hypertension**			
No	0.14 (0.05–0.40)	0.20 (0.07–0.61)	0.31 (0.11–0.91)
Yes	0.15 (0.06–0.40)	0.24 (0.05–0.51)	0.36 (0.09–0.92)
**Diabetes**			
No	0.14 (0.05–0.40)	0.20 (0.07–0.61)	0.32 (0.11–0.91)
Yes	0.14 (0.06–0.26)	0.24 (0.07–1.20)	0.29 (0.16–2.04)
**Allergic rhinitis**			
No	0.15 (0.05–0.41)	0.21 (0.07–0.65)	0.32 (0.11–0.98)
Yes	0.13 (0.06–0.38)	0.19 (0.08–0.51)	0.28 (0.10–0.69)
**Respiratory diseases**			
No	0.14 (0.05–0.4)	0.20 (0.07–0.59)	0.32 (0.11–0.90)
Yes	0.16 (0.07–0.59)	0.26 (0.05–1.08)	0.41 (0.08–1.93)
**Neoplasms**			
No	0.14 (0.06–0.41)	0.21 (0.07–0.61)	0.32 (0.11–0.94)
Yes	0.15 (0.04–0.20)	0.15 (0.07–0.44)	0.29 (0.09–0.75)
**Other chronic diseases**			
No	0.15 (0.06–0.41)	0.20 (0.07–0.61)	0.32 (0.11–0.93)
Yes	0.11 (0.05–0.19)	0.21 (0.09–0.40)	0.32 (0.20–0.52)

**Table 3:** Multivariable analyses (ORs and 95% CI) for the predictors of Ag1 cellular response among 419 vaccinated HCW of CSS, using different outcomes: quintiles of Ag1 (low vs. high, ordinal logistic regression, first column); logarithm of Ag1 (second column); positivity of Ag1 (third column).

	Ag1 Quintiles			Logarithm of Ag1			Ag1 Positivity		
	OR	95% CI	*p*	OR	95% CI	*p*	OR	95% CI	*p*
**Age (year)**	1.02	1.00–1.03	0.124	1.01	0.99–1.03	0.187	1.03	1.01–1.06	0.01
**Gender**									
Female	1			1			1		
Male	0.69	0.43–1.10	0.120	0.80	0.55–1.15	0.221	0.53	0.30–0.94	0.029
**Job profile**									
Nurse	1			1			1		
Physician	1.28	0.77–2.14	0.339	1.08	0.72–1.62	0.718	2.04	1.09–3.82	0.026
Health care assistant (HCA)	1.34	0.67–2.70	0.410	1.11	0.64–1.92	0.713	1.89	0.79–4.48	0.150
Other Clinical staff	1.22	0.74–1.99	0.437	1.07	0.72–1.58	0.742	1.31	0.72–2.40	0.377
Administrative staff	1.76	0.95–3.27	0.073	1.57	0.96–2.55	0.072	1.80	0.86–3.78	0.121
IT/maintenance staff	1.83	0.82–4.12	0.142	1.39	0.72–2.66	0.326	3.16	1.16–8.64	0.025
**Smoking habits**									
Never smokers	1			1			1		
Former smokers	1.02	0.61.63	0.938	0.96	0.66–1.40	0.827	1.01	0.56–1.80	0.986
Current smokers	1.80	1.14–2.85	0.011	1.38	0.96–1.98	0.081	1.93	1.11–3.36	0.019
**BMI**									
Normal weight (BMI18.5-25)	1			1			1		
Underweight (BMI < 18.5)	0.85	0.31–2.31	0.752	0.93	0.42–2.03	0.852	0.97	0.26–3.62	0.958
Overweight (BMI 25–30)	0.99	0.63–1.54	0.968	0.96	0.67–1.37	0.823	1.26	0.73–2.16	0.407
Obesity (BMI > 30)	1.02	0.56–1.84	0.953	1.05	0.65–1.69	0.846	1.10	0.53–2.31	0.791
**ABO blood group**									
O	1			1			1		
A/AB/B	1.50	1.02–2.21	0.041	1.37	1.01–1.86	0.046	1.43	0.90–2.29	0.133
Unknown	0.56	0.33–0.96	0.034	0.70	0.46–1.08	0.101	0.59	0.30–113	0.111
**Autoimmune diseases (ref = NO)**									
Yes	0.62	0.35–1.10	0.103	0.75	0.49–1.16	0.195	0.50	0.25–0.99	0.047
**Immuno-deficiency (ref = NO)**									
Yes	0.83	0.30–2.30	0.719	0.66	0.30–1.46	0.305	1.08	0.31–3.77	0.901
**Hypertension (ref = NO)**									
Yes	0.84	0.50–1.39	0.489	0.87	0.59–1.29	0.496	0.88	0.47–1.62	0.679
**Diabetes (ref = NO)**									
Yes	1.24	0.38–3.99	0.722	1.11	0.43–2.86	0.833	1.12	0.26–4.90	0.880
**Allergic rhinitis (ref = NO)**									
Yes	1.15	0.73–1.81	0.551	1.14	0.78–1.65	0.500	0.96	0.54–1.70	0.896
**Respiratory diseases (ref = NO)**									
Yes	1.33	0.52–3.43	0.551	1.32	0.63–2.76	0.466	1.81	0.56–5.83	0.318
**Neoplasms (ref = NO)**									
Yes	0.63	0.25–1.59	0.325	0.8	0.38–1.68	0.559	1.37	0.44–4.21	0.587
**Other chronic diseases (ref = NO)**									
Yes	0.44	0.15–1.30	0.136	0.7	0.28–1.76	0.452	0.54	0.13–2.15	0.380
**Previous SARS-CoV-2 infection (ref = NO)**									
Yes	3.64	2.28–5.80	<0.001	2.89	2.02–4.13	<0.001	4.24	2.34–7.67	<0.001
**Distance (week) between**									
**vaccination and cellular test**	0.96	0.93–0.99	0.013	0.97	0.95–1.00	0.020	0.94	0.91–0.98	0.003

**Table 4:** Multivariable analyses (ORs and 95% CI) for the predictors of Ag2 cellular response among 419 vaccinated HCW of CSS, using different outcomes: quintiles of Ag2 (low vs. high, ordinal logistic regression, first column); logarithm of Ag2 (second column); positivity of Ag2 (third column).

	Ag2 Quintiles			Logarithm of Ag2			Ag2 Positivity		
	OR	95% CI	*p*	OR	95% CI	*p*	OR	95% CI	*p*
**Age (year)**	1.02	1.00–1.04	0.055	1.01	0.99–1.03	0.264	1.03	1.00–1.05	0.018
**Gender**									
Female	1			1			1		
Male	0.98	0.61–1.57	0.926	0.87	0.59–1.27	0.459	1.01	0.58–1.77	0.962
**Job profile**									
Nurse	1			1			1		
Physician	1.18	0.70–1.97	0.535	1.06	0.69–1.62	0.800	1.64	0.88–3.07	0.119
Health care assistant (HCA)	0.96	0.48–1.95	0.917	0.94	0.52–1.67	0.823	1.03	0.44–2.44	0.944
Other Clinical staff	1.28	0.78–2.08	0.327	1.13	0.75–1.70	0.573	1.55	0.85–2.83	0.156
Administrative staff	1.71	0.92–3.18	0.088	1.55	0.93–2.60	0.093	2.55	1.19–5.49	0.017
IT/maintenance staff	1.76	0.78–3.98	0.175	1.47	0.74–2.92	0.270	1.84	0.68–4.96	0.228
**Smoking habits**									
Never smokers	1			1			1		
Former smokers	1.13	0.69–1.83	0.627	1.06	0.71–1.58	0.768	1.02	0.57–1.83	0.934
Current smokers	1.63	1.04–2.55	0.034	1.41	0.96–2.06	0.076	2.02	1.15–3.56	0.015
**BMI**									
Normal weight (BMI 18.5–25)	1			1			1		
Underweight (BMI < 18.5)	0.83	0.30–2.32	0.719	0.93	0.41–2.13	0.871	1.09	0.31–3.92	0.890
Overweight (BMI 25–30)	0.98	0.63–1.52	0.926	0.96	0.66–1.40	0.841	1.18	0.69–2.04	0.547
Obesity (BMI > 30)	1.10	0.61–1.99	0.753	1.09	0.66–1.80	0.746	1.45	0.67–3.10	0.343
**ABO blood group**									
O	1			1			1		
A/AB/B	1.76	1.19–2.59	0.004	1.54	1.11–2.13	0.009	2.49	1.55–4.01	<0.001
Unknown	0.59	0.34–1.01	0.055	0.72	0.46–1.11	0.380	0.63	0.33–1.19	0.155
**Autoimmune diseases (ref = NO)**									
Yes	0.69	0.39–1.20	0.184	0.75	0.48–1.18	0.201	0.56	0.29–1.08	0.086
**Immuno-deficiency (ref = NO)**									
Yes	0.75	0.27–2.05	0.575	0.70	0.30–1.62	0.400	1.14	0.34–3.87	0.828
**Hypertension (ref = NO)**									
Yes	0.81	0.48–1.35	0.412	0.88	0.58–1.34	0.557	0.84	0.45–1.54	0.568
**Diabetes (ref = NO)**									
Yes	1.62	0.46–5.70	0.449	1.21	0.45–3.29	0.704	1.20	0.29–5.03	0.801
**Allergic rhinitis (ref = NO)**									
Yes	1.18	0.74–1.86	0.491	1.07	0.72–1.58	0.746	1.29	0.73–2.28	0.378
**Respiratory diseases (ref = NO)**									
Yes	1.22	0.45–3.28	0.697	1.28	0.59–2.78	0.539	1.19	0.39–3.68	0.758
**Neoplasms (ref = NO)**									
Yes	0.55	0.22–1.36	0.193	0.83	0.38–1.80	0.632	0.45	0.15–1.37	0.159
**Other chronic diseases (ref = NO)**									
Yes	0.71	0.24–2.10	0.534	0.71	0.27–1.86	0.480	1.42	0.33–6.15	0.637
**Previous SARS-CoV-2 infection (ref = NO)**									
Yes	3.32	2.07–5.33	<0.001	2.68	1.84–3.91	<0.001	3.05	1.67–5.55	<0.001
**Distance (week) between**									
**vaccination and cellular test**	0.96	0.93–0.99	0.014	0.97	0.94–0.99	0.014	0.98	0.94–1.01	0.232

**Table 5:** Multivariable analyses (ORs and 95% CI) for the predictors of Ag3 cellular response among 419 vaccinated HCW of CSS, using different outcomes: quintiles of Ag3 (low vs. high, ordinal logistic regression, first column); logarithm of Ag3 (second column); positivity of Ag3 (third column).

	Ag3 Quintiles			Logarithm of Ag3			Ag3 Positivity		
	OR	95% CI	*p*	OR	95% CI	*p*	OR	95% CI	*p*
**Age (year)**	1.02	1.00–1.04	0.027	1.02	1.00–1.03	0.048	1.04	1.01–1.06	0.005
**Gender**									
Female	1			1			1		
Male	0.86	0.54–1.36	0.511	0.78	0.53–1.14	0.192	0.51	0.29–0.92	0.024
**Job profile**									
Nurse	1			1			1		
Physician	1.13	0.68–1.87	0.647	1.04	0.68–1.59	0.850	2.17	1.11–4.24	0.024
Health care assistant (HCA)	1.22	0.62–2.43	0.562	0.98	0.55–1.74	0.944	1.47	0.58–3.73	0.418
Other Clinical staff	1.33	0.81–2.18	0.267	1.13	0.75–1.70	0.559	1.59	0.84–3.01	0.155
Administrative staff	2.08	1.12–3.84	0.020	1.62	0.98–2.70	0.062	2.52	1.09–5.80	0.030
IT/maintenance staff	1.62	0.72–3.66	0.248	1.44	0.73–2.85	0.289	2.6	0.88–7.72	0.084
**Smoking habits**									
Never smokers	1			1			1		
Former smokers	0.96	0.61–1.60	0.954	0.98	0.66–1.46	0.939	0.66	0.35–1.21	0.179
Current smokers	1.43	0.91–2.25	0.116	1.38	0.95–2.01	0.093	1.14	0.63–2.04	0.671
**BMI**									
Normal weight (BMI 18.5–25)	1			1			1		
Underweight (BMI < 18.5)	0.71	0.26–1.94	0.501	0.79	0.35–1.78	0.563	0.82	0.23–3.02	0.771
Overweight (BMI 25–30)	1.24	0.80–1.93	0.339	1.10	0.76–1.59	0.625	1.86	1.02–3.38	0.042
Obesity (BMI > 30)	1.22	0.67–2.23	0.507	1.08	0.65–1.77	0.772	2.15	0.91–5.08	0.081
**ABO blood group**									
O	1			1			1		
A/AB/B	1.60	1.09–2.35	0.016	1.46	1.06–2.02	0.021	1.37	0.83–2.27	0.219
Unknown	0.57	0.33–0.98	0.044	0.68	0.44–1.05	0.081	0.60	0.30–1.18	0.137
**Autoimmune diseases (ref = NO)**									
Yes	0.67	0.38–1.18	0.164	0.75	0.48–1.17	0.206	0.32	0.16–0.65	0.001
**Immuno-deficiency (ref = NO)**									
Yes	0.55	0.20–1.54	0.256	0.64	0.28–1.47	0.292	1.12	0.33–3.86	0.854
**Hypertension (ref = NO)**									
Yes	0.85	0.51–1.42	0.544	0.88	0.58–1.32	0.536	0.76	0.39–1.48	0.421
**Diabetes (ref = NO)**									
Yes	1.32	0.38–4.61	0.665	1.57	0.58–4.21	0.373	2.53	0.44–14.6	0.299
**Allergic rhinitis (ref = NO)**									
Yes	0.92	0.58–1.47	0.742	0.91	0.62–1.34	0.643	1.01	0.55–1.85	0.981
**Respiratory diseases (ref = NO)**									
Yes	1.62	0.59–4.44	0.352	1.41	0.65–3.04	0.387	0.96	0.29–3.23	0.946
**Neoplasms (ref = NO)**									
Yes	0.67	0.27–1.65	0.379	0.85	0.39–1.84	0.682	0.46	0.15–1.45	0.187
**Other chronic diseases (ref = NO)**									
Yes	0.73	0.23–2.29	0.588	0.71	0.27–1.84	0.474	1.41	0.27–7.38	0.683
**Previous SARS-CoV-2 infection (ref = NO)**									
Yes	3.21	2.02–5.12	<0.001	2.90	2.00–4.21	<0.001	3.04	1.53–6.03	<0.001
**Distance (week) between**									
**vaccination and cellular test**	0.96	0.93–0.99	0.016	0.97	0.95–1.00	0.051	0.95	0.92–0.99	0.026

## Data Availability

The data presented in this study are available within the article and Supplementary Materials.

## Supplementary Materials

The following supporting information can be downloaded at: https://www.mdpi.com/article/10.3390/vaccines10071031/s1. Table S1: Cut-off values of the distribution of each Ag among the 419 HCW of CSS; Table S2: Summary of the direction of the associations between selected socio-demographic and clinical characteristics and levels of serological response (first column, Costa 2022) and of T-cell response for each Ag (second-fourth columns) in vaccinated HCW of CSS; Table S3: Number of subjects (First Column) and prevalence of positivity for each antigenic stimuli (Ag) among 419 vaccinated HCW of CSS by distance between vaccination and T-Cell immunity test, stratified by previous SARS-CoV2 infection; Figure S1. Results of multivariable ordinal logistic regression model (ORs and 95% CI) for predictors of higher Ag1 values (quintiles of distribution) among 419 vaccinated HCW of CSS; Figure S2. Results of multivariable ordinal logistic regression model (ORs and 95% CI) for predictors of higher Ag2 values (quintiles of distribution) among vaccinated HCW of CSS; Figure S3. Results of multivariable ordinal logistic regression model (ORs and 95% CI) for predictors of higher Ag3 values (quintiles of distribution) among vaccinated HCW of CSS.
