# Supplementary material for: Cellular Immune Response to BNT162b2 mRNA COVID-19 Vaccine in a Large Cohort of Healthcare Workers in a Tertiary Care University Hospital
Source: Vaccines (Basel). 2022 Jun 27;10(7):1031. doi: 10.3390/vaccines10071031 (PMC9316283; doi:10.3390/vaccines10071031)
Supplement: Supplementary file 1 [file vaccines-10-01031-s001.zip › vaccines-1745670-supplementary.pdf]

Supplementary Materials

Table S1. Cut-off values of the distribution of each Ag among the 419 HCW of CSS.

Ag1 (quintile cut-off) IU/ml	Ag2 (quintile cut-off) IU/ml	Ag3 (quintile cut-off) IU/ml
<=0.04	<=0.05	<=0.09
0.05-0.10	0.06-0.14	0.10-0.21
0.11-0.20	0.15-0.30	0.22-0.48
0.21-0.49	0.31-0.80	0.49-1.34
>= 0.50	>=0.81	>=1.35

Table S2. Summary of the direction of the associations between selected socio-demographic and clinical characteristics and levels of serological response (first column, Costa 2022) and of T-cell response for each Ag (second-fourth columns) in vaccinated HCW of CSS.

	Serological response	Ag1	Ag2	Ag3
Older age	↓	↑	↑	↑
Male sex	↓	↓	↓↑	↓
Current smoking (vs never)	↓	↑	↑	↑
Overweight-obesity (vs normal)	=↑	=↑	=↑	↑↑
Blood Groups A/AB/B (vs 0)	=*	↑	↑	↑
Previous SARS-CoV-2 infection	↑	↑	↑	↑
Autoimmune disease	=↓	↓	↓	↓
Immuno-deficiency	↓	↓↑	↓↑	↓↑
Increasing time elapsed between vaccination and immunity test	↓	↓	↓	↓

↑: increased response; ↓: reduced response; ↓↑: for Ag, slightly inconsistent results between the outcomes evaluated (quintiles of distribution, log-transformed Ag values, Ag positivity) In **red bold:** p< 0.05. For Ag, at least one association with p< 0.05 among the outcomes evaluated (quintiles of distribution, log-transformed Ag values, Ag positivity, see Tables 3,4, and 5). * single blood groups were analysed, with inconsistent and not statistically significant associations.

Table S3. Number of subjects (First Column) and prevalence of positivity for each antigenic stimuli (Ag) among 419 vaccinated HCW of CSS by distance between vaccination and T-Cell immunity test, stratified by previous SARS-CoV-2 infection.

	N of subjects	Ag1 Positivity N (%)	Ag2 Positivity N (%)	Ag3 Positivity N (%)
Subjects WITHOUT previous SARS-CoV-2 Infection	*337*			
**Time elapsed (months) between vaccination and T-cell test**				
<5	18	7 (38.9%)	10 (55.6%)	10 (55.6%)
>=5 - <6	96	51 (53.1%)	56 (58.3%)	69 (71.9%)
>=6 - <7	37	23 (62.2%)	26 (70.3%)	27 (73.0%)
>=7 - <8	108	44 (40.7%)	56 (51.9%)	66 (61.1%)
>=8	78	23 (29.5%)	38 (48.7%)	45 (57.7%)

Subjects WITH previous SARS-CoV-2 Infection	*82*			
**Time elapsed (months) between vaccination and T-cell test**				
<5	8	8 (100.0%)	7 (87.5%)	8 (100.0%)
>=5 - <6	30	22 (73.3%)	23 (76.7%)	26 (86.7%)
>=6 - <7	5	4 (80.0%)	4 (80.0%)	4 (80.0%)
>=7 - <8	28	21 (75.0%)	21 (75.0%)	24 (85.7%)
>=8	11	6 (54.6%)	7 (63.6%)	7 (63.6%)

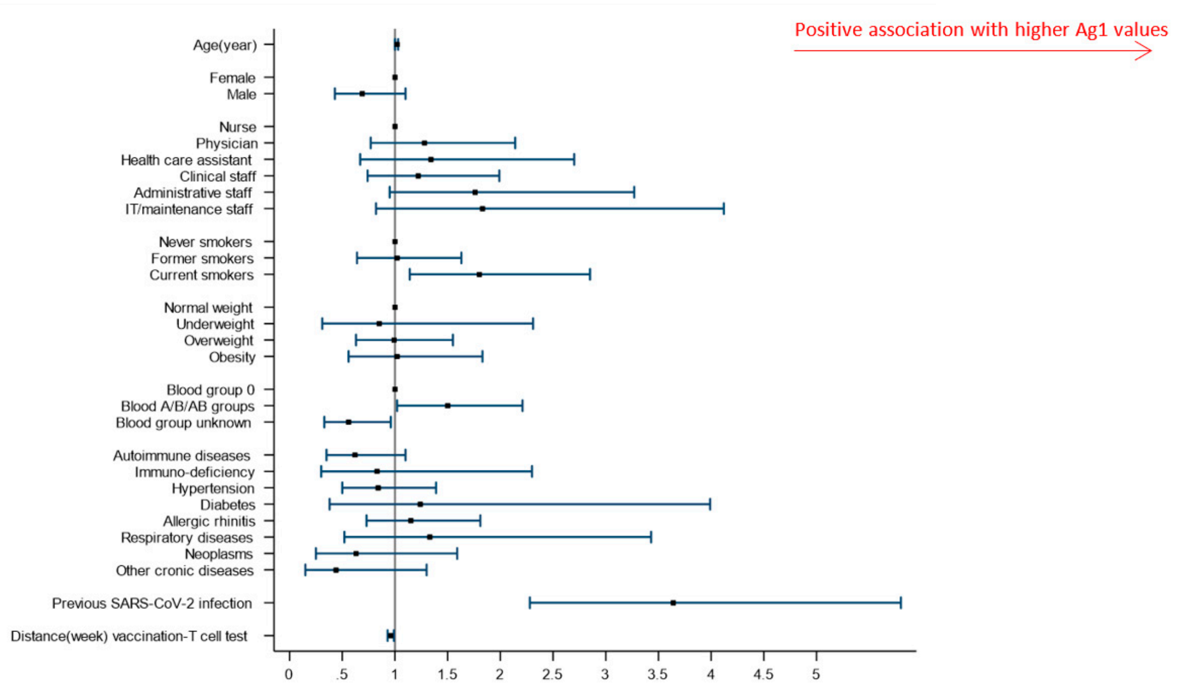

Figure S1. Results of multivariable ordinal logistic regression model (ORs and 95% CI) for predictors of higher Ag1 values (quintiles of distribution) among 419 vaccinated HCW of CSS.

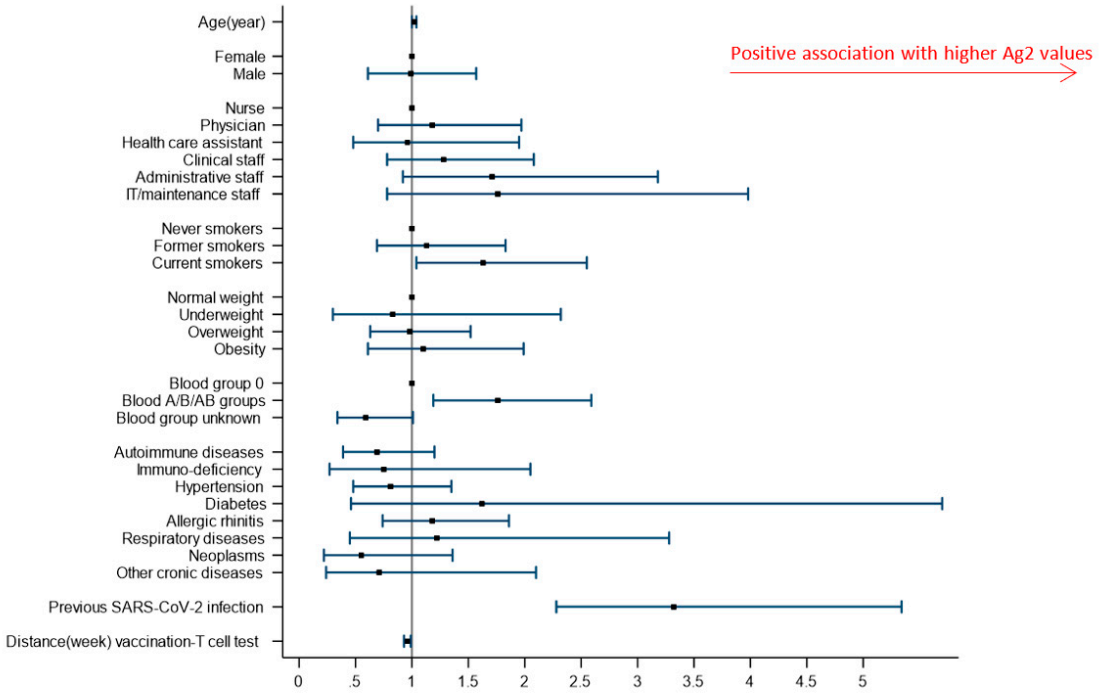

Figure S2. Results of multivariable ordinal logistic regression model (ORs and 95% CI) for predictors of higher Ag2 values (quintiles of distribution) among vaccinated HCW of CSS.

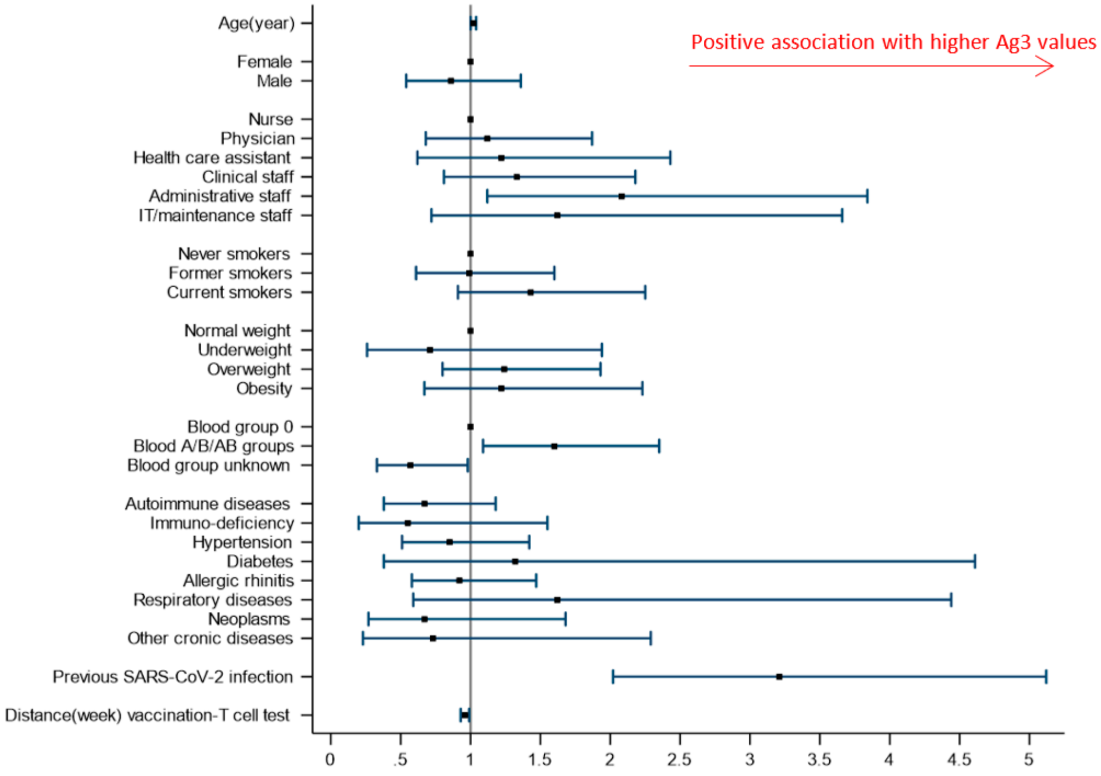

Figure S3. Results of multivariable ordinal logistic regression model (ORs and 95% CI) for predictors of higher Ag3 values (quintiles of distribution) among vaccinated HCW of CSS.
